# Development and Engineering Application of a Vertically Installed FBG Sensor for Subgrade Strain Monitoring

**DOI:** 10.3390/mi17101112

**Published:** 2026-09-23

**Authors:** Jianping He, Haigang Wang, Tongchun Qin, Di Ning

**Affiliations:** 1 National Field Observation and Research Station (Hebei Cangzhou) for Groundwater and Land Subsidence, Cangzhou 061000, China; 2 School of Civil Engineering, Nantong Institute of Technology, Nantong 226000, China; 3 College of Resource Environment and Tourism, Capital Normal University, Beijing 100048, China

**Keywords:** vertically installed FBG sensor, vertical deformation, Saint-Venant effect, local compressive strain measurement, field test

## Abstract

Subgrade deformation under load is accumulated from the local compressive strain of each layer. Solely relying on the overall settlement monitoring at the top surface makes it difficult to reveal the internal strain distribution and the evolutionary characteristics of early damage. Therefore, acquiring the local compressive strain field along the depth direction is of great engineering significance for subgrade condition assessment and safety early warning. In this paper, a vertically installed FBG sensor (VI-FBG) with GFRP packaging suitable for layered strain measurement of subgrade is developed. The effectiveness of the sensor is verified by direct compression tests and compressive strain tests with the sensor embedded in concrete. Furthermore, the sensor was buried in the subgrade of a high-speed railway to carry out strain monitoring. The test results show that the sensor has favorable sensing performance, with a strain sensitivity of 1.2 pm/με and a temperature sensitivity of 16.84 pm/°C. The measured compressive strain of concrete is in good agreement with the theoretical value, and the relative error is less than 8%. The field monitoring data can truly reflect the vertical compressive strain during subgrade filling. The measured time-history curve exhibits good consistency with the displacement time-history curve obtained by the adjacent FBG settlement sensor. This study verifies that the sensor is applicable to the monitoring of local vertical compressive deformation of subgrade, and its GFRP encapsulation endows it with good engineering applicability.

## 1. Introduction

With the rapid development of transportation infrastructure, high-speed railways, expressways, and urban roads are increasingly being constructed in hilly regions characterized by complex terrain and geological conditions. As the primary load-bearing structure of transportation pavement systems, subgrades are susceptible to differential settlement, plastic deformation, and localized subsidence during construction and long-term operation. These defects can impair riding smoothness and passenger comfort on highways and urban roads, while posing a serious threat to the safe and stable operation of high-speed railways, which are subject to stringent deformation-control requirements. A subgrade consists of multiple compacted fill layers. Under the combined effects of train-induced dynamic loads, fill self-weight, groundwater, and environmental temperature variations, vertical compressive deformation develops within the subgrade. Conventional settlement sensors generally measure only the total settlement at discrete points, making it difficult to capture local compressive strains at different depths or identify the soil layers responsible for settlement. Because the macroscopic settlement of a subgrade fundamentally results from the layer-by-layer accumulation of vertical local compressive strains, surface settlement data alone cannot adequately characterize the internal, layer-specific compression behavior or distinguish the contribution of individual soil layers. Nor can such data capture small, abrupt local strain changes caused by insufficient compaction, weak interlayers, or other concealed internal defects. Consequently, it remains difficult to reveal the distribution of internal strains throughout the entire construction and service life of a subgrade or to clarify the mechanisms governing early damage evolution. Long-term, real-time, and high-precision monitoring of layer-specific deformation and internal local compressive strain is therefore of great engineering significance for elucidating subgrade deformation mechanisms, enabling early safety warnings, and advancing refined assessments of in-service subgrade performance.

At present, subgrade monitoring mainly focuses on settlement measurement. Subgrade settlement monitoring technology has undergone decades of development, and various monitoring methods and sensing technologies have been continuously developed and applied in engineering practice, forming a technical system integrating traditional electromechanical sensing and modern optical sensing, which enables deformation monitoring of both the surface and deep strata of subgrades. As conventional settlement monitoring devices, settlement plates are easy to operate and cost-effective, yet suffer from low monitoring efficiency, discontinuous data acquisition and poor real-time performance. In addition to manual settlement monitoring with settlement plates, large-scale measurement technologies such as the unmanned aerial vehicle (UAV), the Global Positioning System (GPS) and Interferometric Synthetic Aperture Radar (InSAR) can realize millimeter-level ground settlement monitoring, but their monitoring costs are relatively high [1,2,3,4]. When fiber Bragg grating or resistive displacement sensors are applied to pavement and subgrade settlement monitoring, multiple sensors can be arranged in the same borehole to achieve multi-point displacement measurement in deep soil with a precision up to the submillimeter level. However, such methods are associated with high layout costs and significant systematic errors caused by excessively long traction lines [5,6,7,8]. Distributed optical fiber sensing technology has the remarkable advantage of enabling long-distance and continuous structural monitoring, mainly including distributed optical time-domain reflectometry (OTDR), distributed Brillouin optical fiber sensing (BOTDR/BOTDA) and distributed fiber vibration sensing (DVS/DAS), which have been widely applied in fields such as oil pipelines, bridges and tunnels [9,10,11,12]. In the field of settlement monitoring, Zheng et al. developed a novel spring-shaped fiber-optic displacement sensor based on OTDR technology, with a measurement range of up to 90 mm and a minimum displacement resolution of 0.173 mm [13]. Shi, Xue and other researchers applied distributed Brillouin optical fiber sensing technology to pavement settlement monitoring, and calculated the vertical settlement deformation from longitudinal strain by arranging distributed optical fiber sensors along the length of the subgrade [14,15,16]. Furthermore, Shi and Wang installed sensors through boreholes and realized continuous deformation monitoring of deep soil displacement up to hundreds of meters based on weak fiber Bragg grating and distributed Brillouin optical fiber sensing technologies [17,18]. Aiming at the key problem of subgrade uneven settlement caused by underground soil cavity collapse, He et al. adopted distributed fiber-optic vibration and strain sensors to simultaneously monitor the vibration signals of soil cavity collapse and soil settlement deformation, realizing the full-process monitoring of subgrade collapse and settlement in karst-developed areas and greatly improving the early warning capability for subgrade disasters [19].

The above sensing technologies mainly focus on the settlement and displacement monitoring at the surface or inside of the subgrade/soil mass, and it is difficult to reveal the internal deformation evolution mechanism of soil and capture the soil compaction and creep processes. In view of the above research status, based on the previous achievements of our research group [20], this study further optimizes the design of the vertically installed fiber Bragg grating sensor and expands its application research on vertical strain monitoring of subgrades. Firstly, finite element analysis is carried out on sensors with three GFRP encapsulation structures of different length–diameter ratios, and the optimal GFRP structural dimensions are determined according to the grating strain curves and GFRP stress nephograms. Secondly, static pressure tests and temperature tests are conducted to investigate the sensor characteristics, including the sensing coefficient and hysteresis performance. Thirdly, uniaxial compression tests on C50 concrete specimens are performed to verify the effectiveness of the sensor in measuring compressive strain inside concrete structures. Finally, field monitoring tests are carried out on a high-speed railway subgrade in the mountainous area of Southwest China. The developed sensor is embedded in the subgrade bottom to perform local vertical compressive strain monitoring during the subgrade filling stage within about four months, which verifies the engineering applicability and reliability of the sensor in practical engineering.

## 2. Optimized Design of the Vertically Installed FBG Sensor

### 2.1. Principle of Fiber Bragg Grating Sensing Technology

A FBG is a kind of optical sensing element formed by introducing a periodic refractive index modulation in the fiber core. Its basic working principle follows the Bragg phase-matching condition:

(1) $$\lambda_{B}=2n_{eff}\wedge$$

where $\lambda_{B}$ is the Bragg wavelength, $n_{eff}$ is the effective refractive index of the fiber core, and ∧ is the grating period.

When broadband light is injected into the optical fiber, the FBG will selectively reflect a narrowband light whose wavelength satisfies the Bragg condition. When the FBG is subjected to external physical quantities such as strain, temperature, pressure, or displacement, both $n_{eff}$ and ∧ will change accordingly, resulting in a shift of the reflected Bragg wavelength. By demodulating and measuring the Bragg wavelength shift $\lambda_{B}$ with an optical interrogator, the variation of the measured physical parameters can be accurately obtained. Previous studies have shown that the Bragg wavelength shift exhibits the following linear relationship with strain and temperature.

(2) $$\Delta \lambda_{B}=C_{\varepsilon }\Delta \varepsilon +C_{T}\Delta T$$

where $\Delta \lambda_{B}$ is the central wavelength variation of the FBG; Δε,ΔT are the strain and temperature variations, respectively; $C_{\varepsilon }\;\left(C_{\varepsilon }=1.2\;\mathrm{pm}/\mu \varepsilon \right)$ and $C_{T}\;\left(C_{T}=10.8\;\mathrm{pm}/{}^{\circ}C\right)$ are the strain and temperature sensitivity coefficients, respectively [21,22].

Equation (3) is the sensing equation for the FBG temperature sensor, which is used for temperature compensation for strain or other FBG sensors.

(3) $$\Delta \lambda_{T}=C_{TT}\Delta T$$

Based on Equations (2) and (3), the temperature-independent strain can be calculated with Equation (4).

(4) $$\Delta \varepsilon =\left(\Delta \lambda_{B}-\phi \Delta \lambda_{TT}\right)/C_{\varepsilon }$$

where $\phi =C_{T}/C_{TT}$. If the temperature sensor and strain sensor adopt the same packaging materials, their temperature sensing coefficients are equal, then ϕ=1.

### 2.2. Optimized Design of VI-FBG Sensor and Sensing Performance

**(1) Sensor Design**

Figure 1 shows the design drawing of the sensor, which is mainly composed of a stainless-steel upper cover, a stainless-steel bottom base, an FRP encapsulation cylinder, an FBG sensing element, and an optical fiber cable. The FBG is embedded in the center of the central axis of the GFRP cylinder by a pultrusion molding process. It can be seen that the bottom base includes two cylinders, and their diameters are 32 mm and 20 mm, respectively; the total height is 13 mm; the diameter of the upper cover is 32 mm. The total length of the GFRP was fixed at L = 38 mm, while the GFRP diameter was varied as D = 3, 5, and 7 mm, corresponding to L/D = 12.67, 7.6, and 4.43, respectively.

To determine the appropriate GFRP dimensions, a three-dimensional finite-element parametric analysis was conducted using SOLID186 elements as shown in Figure 2. The maximum von Mises stresses of the complete sensor were 15.61, 5.73, and 3.22 MPa for D = 3, 5, and 7 mm, respectively. The von Mises stress distributions along the sensor axis showed that the central GFRP region reached an approximately stable stress state away from the end interfaces, while the D = 3 mm configuration exhibited the most pronounced end-effect-induced variation, as shown in Figure 3. Here, the bottom boundary of the sensor model was fully constrained in all displacement directions, the lateral boundaries were defined as free boundaries, and a uniform vertical compressive load was applied on the top surface to simulate its actual service mechanical state. Both the GFRP encapsulation and stainless-steel base of the sensor adopted linear elastic constitutive models, and bonded contact was set for all internal interfaces to ensure coordinated structural deformation under compression.

The axial strain deviation in the grating region was also evaluated using the dimensionless parameters L/D and dg/D, where dg denotes the distance from the center of the grating region to the nearest end face. The maximum axial strain deviations for D = 3, 5, and 7 mm were 0.001%, 0.248%, and 1.421%, respectively, as shown in Figure 4. A strain deviation below 5% was adopted as the criterion for negligible Saint-Venant end effects. The results demonstrate that the selected GFRP diameter of 5 mm and length-to-diameter ratio of L/D = 7.6 satisfy this criterion and provide a suitable balance between end-effect suppression, mechanical stability, and sensor compactness.

Figure 5 shows the influence of the GFRP diameter on the strain distribution within the FBG sensing region. The 15 mm FBG grating region was positioned at the center of the GFRP, from z = 24.5 to 39.5 mm. The axial strain at each axial station was obtained by averaging the axial strains of all GFRP nodes across the complete cross-section. It can be seen that the axial strain remains nearly constant throughout the grating region for all three diameters. The mean axial strains were −76.70 με, −27.07 με and −13.61 με for D = 3, 5, and 7 mm, respectively. The maximum deviations from the corresponding mean values were 0.1891%, 0.0665%, and 0.0636%, respectively. All deviations are well below the 5% criterion used to identify negligible Saint-Venant end effects. Comparative numerical simulations were performed on GFRP encapsulations with diameters of 4 mm, 5 mm, and 6 mm. The simulation results confirm that the sensor with a 5 mm diameter achieves uniform strain distribution, stable linear response, and superior strain transfer efficiency, which is determined to be the optimal structural configuration.

**(2) Experimental Study on Sensing Performance**

Figure 6 shows the physical drawing of the VI-FBG sensor. The FRP encapsulation cylinder is made of glass fiber-reinforced polymer (GFRP) with a length of 38 mm and a diameter of 5 mm. To ensure uniform loading of the entire GFRP component within a structure such as concrete, a stainless-steel cylinder was sleeved outside the FRP to prevent direct contact between the FRP column and concrete. The stainless-steel cylinder has a length of 26 mm. This design guarantees that the cylinder merely serves to protect the GFRP column without undertaking vertical load.

Figure 7 shows the experimental setup for the strain and temperature sensing performance of the VI-FBG sensor. A high-precision testing machine with a displacement accuracy of 0.001 mm was used as the strain loading equipment, and the strain values were calculated from the displacement of the testing machine. Prior to the formal test, the sensor was preloaded to 10 N to eliminate errors induced by gaps between the testing machine and the sensor. A conventional high-temperature chamber was adopted as the temperature test device. During the test, the displacement step and temperature step were 0.003 mm (about 80 με) and 10 °C, respectively. Two sets of strain calibration tests were performed, one of which was a cyclic loading–unloading test, and the test results are presented in Figure 8. It can be seen that the strain sensing coefficient is about 1.2 pm/με. Based on the cyclic loading–unloading test data, the hysteresis behavior and measurement repeatability can be quantitatively obtained and analyzed. It can be seen that both the loading and unloading curves exhibited a good linear relationship. The loading and unloading curves are nearly coincident, and the wavelength variation over the full measurement range is:

(5) $$\Delta \lambda_{\mathrm{FS}}=1558.8934-1557.7438=1.1496\;\mathrm{nm}$$

The maximum difference between the loading and unloading curves over the common strain range was 0.02915 nm. The corresponding hysteresis error was:

(6) $$e_{hys}=\frac{0.02915}{1.1496}\times 100\%=2.54\%\;\;\;\;\;\;\;\;\;\;$$

After unloading, the wavelength differed from the initial value by 0.0213 nm, corresponding to a return-point repeatability error of 1.85% of the full-scale response.

Figure 9 is the hysteresis error curve of Test II. The hysteresis error curve shows that the loading and unloading responses are generally in good agreement over the investigated strain range. The maximum wavelength difference between the loading and unloading paths was 0.0291 nm, corresponding to a maximum hysteresis error of 2.54% of the full-scale wavelength response. Most of the hysteresis error remained below 0.005 nm in the intermediate strain range, indicating stable and nearly reversible sensor behavior. A relatively larger deviation was observed near −883 με and at the return point. These local deviations may be associated with the limited number of discrete data points, interpolation between loading and unloading measurements, and slight changes in the contact or seating conditions during unloading. Because only one loading–unloading cycle was performed, the obtained value should be regarded as a preliminary single-cycle hysteresis indicator. Additional repeated cycles are required for a statistical characterization of hysteresis and repeatability. Figure 10 shows the test result of temperature sensing performance; the temperature sensing coefficient is about 16.84 pm/°C.

## 3. Compression Test of Concrete Specimens Based on VI-FBG Sensor

With a total height of only 53 mm, the VI-FBG sensor can be embedded in concrete or asphalt pavements for layered strain monitoring, which is one of its typical application scenarios. To verify the effectiveness of this application scenario, a normal pressure test on concrete blocks was designed in this study. One VI-FBG sensor and one temperature FBG sensor were installed at the center of the concrete block to measure strain and temperature under normal pressure loading. For comparative study, one bare fiber Bragg grating (FBG) with an initial central wavelength of 1535.4387 nm was bonded onto the concrete surface. The concrete specimen has a length of 100 mm, a width of 100 mm and a height of 300 mm. Figure 11 shows the layout of sensors embedded in concrete and their arrangement inside the mould; the three sensors are located at the same elevation.

Figure 12 shows the concrete loading test system, which consists of a fiber Bragg grating demodulator, a material testing machine and a concrete specimen. The load was applied uniformly at a rate of 2 kN/min until the maximum load of 136 kN was reached. Figure 13 shows the wavelength changes of the FBG-T sensor. As observed, the central wavelength of the temperature sensor hardly varies at each load level, which demonstrates that the concrete internal temperature remains constant during loading. Thus, temperature compensation is unnecessary for the strain readings of VI-FBG and bare FBG sensors. Here, the temperature sensor FBG-T also adopts a GFRP-packaged bare FBG, and the diameter of the GFRP is 5 mm, which is sleeved with a stainless-steel tube to ensure that FBG-T remains in a free state inside the stainless-steel tube, so the temperature sensing coefficient is 16.84 pm/°C.

In this design, the length of the stainless-steel tube is shorter than that of the exposed GFRP column. So, the stainless-steel tube undertakes an extremely small axial load during compression. Instead of exhibiting strictly zero strain, its axial strain contribution is reasonably assumed to be negligible in the present analysis for simplification purposes. For comparison with the experimental results, the strain values at the center position of the sensor under different loads were calculated using the following formulas. Based on the deformation compatibility condition, the axial strains of GFRP and concrete are equal, expressed as:

(7) $$\varepsilon_{GFRP}=\varepsilon_{concrete}=\varepsilon$$

The total axial force is jointly resisted by the three constituent materials:

(8) $$F_{total}=F_{GFRP}+F_{concrete}$$

Substituting the stress–strain relationship (*σ* = *Eε*) and force equilibrium (*F* = *σA*) into the equation yields:

(9) $$F_{total}=\varepsilon \left(E_{GFRP}A_{GFRP}+E_{concrete}A_{concrete}\right)$$

Here, *ε* is the axial strain; *F* is the axial force; *E* is the modulus of elasticity; *A* is the cross-sectional area.

Furthermore, the total axial stiffness of the composite system is the sum of the individual stiffnesses:

(10) $$K_{total}=K_{FRP}+K_{concrete}$$

The elastic moduli of the concrete specimen and FRP are known to be 34,500 MPa and 45,000 MPa, respectively. Therefore, the strain value of the sensor can be easily calculated according to Equation (9) when the normal pressure is known.

Figure 14 shows the wavelength changes of VI-FBG and bare FBG sensors under each load. Figure 15 shows the strains calculated from the wavelength changes of VI-FBG and bare FBG sensors and relative errors with respect to the theoretical strain values. It can be seen that the central wavelength of the VI-FBG sensor and the bare FBG sensor have a good linear relationship with the load when the concrete beam is in the elastic stage, and the relative errors between the strain measured by VI-FBG and the theoretical strain at all load points are controlled within 8% except for the first load level. It can be further observed that the strain values measured by VI-FBG are larger than those measured by bare FBG. This is mainly attributed to the friction constraint from the loading platens at both ends of the specimen, which produces lateral confinement on the surface concrete and restrains vertical compressive deformation. In contrast, the central region is free from boundary friction and undergoes greater compressive deformation. When the load reached 78.56 kN, the bare FBG was severed by burrs on the concrete surface, terminating subsequent strain acquisition.

## 4. Case Study: Vertical Strain Monitoring of High-Speed Railway Subgrade Based on the VI-FBG Sensor

As the main load-bearing structure for operating vehicles in transportation systems, the smoothness of high-speed railway subgrades is crucial to ensuring the safe operation of high-speed railways. Carrying out deformation monitoring of high-speed railway subgrades has important theoretical significance and engineering practical value: it enables the timely mastery of the evolution law of subgrade deformation, the early warning of potential defects, and provides guidance for subgrade construction, maintenance, repair and reinforcement treatment, thus safeguarding the long-term safe and stable operation of high-speed railways, improving the service performance of lines and extending the service life of engineering structures. To this end, we adopted a solar power supply system and carried out vertical strain monitoring during the subgrade filling period by installing relevant sensors at the subgrade bottom of a high-speed railway under construction in the mountainous region of Southwest China. The detailed sensor layout procedures are as follows, and the corresponding layout is shown in Figure 16.

**Step 1**: Excavate a cubic trench with a depth of 20 cm and both length and width of 20 cm at the preset sensor layout position.

**Step 2**: Place a stainless-steel bottom base plate with a side length of 15 cm into the cubic trench.

**Step 3**: Fix the VI-FBG sensor onto the bottom base plate with AB glue, and arrange the FBG temperature sensor (named as TFBG) adjacent to the VI-FBG sensor simultaneously.

**Step 4**: Cover the top of the VI-FBG sensor with an upper base plate.

**Step 5**: Backfill the sensor with the excavated filler, and route the transmission lines out of the trench to the integration box along the excavated groove.

The bottom base plate is designed to ensure that the VI-FBG sensor remains perpendicular to the subgrade surface at all times, while the upper base plate is used to protect the sensor from damage during the subgrade compaction process. The FBG-T sensor is applied for temperature compensation of the VI-FBG sensor. A wireless FBG demodulator (Model BRX-WSFA8) produced by Dalian Boruixin Technology Co., Ltd.,Dalian, China, was selected as the sensor data acquisition instrument, with a demodulation accuracy of ±1.5 pm, powered by solar energy and a data sampling frequency of 4 times per day. At this monitoring point, the initial central wavelengths of the VI-FBG and FBG-T sensors were 1545.3476 nm and 1542.0187 nm, respectively.

The monitoring period lasted from 18 September 2025 to 21 January 2026, and the subgrade filling height was 4.35 m. It took 45 days for the subgrade to be filled to the design height of 4.35 m. Figure 17 presents the time-series data monitored by the FBG-T temperature sensor (it can also be packaged in the GFRP). It can be seen that the internal temperature of the subgrade is affected by the external ambient temperature. Therefore, temperature compensation is required for the VI-FBG sensor. Figure 18 shows the time-series wavelength data of the VI-FBG sensor before and after temperature compensation.

Figure 19 presents the curves of cumulative vertical strain and single-event strain variation measured by the subgrade filling sensors. It can be seen that the cumulative strain curve became generally flat, indicating that the sensor was less disturbed by external loads after 16 November 2025. By 21 January 2026, the maximum cumulative strain value reached −770.28 με. It can be seen from Figure 19 and Figure 20 that, without temperature compensation for the VI-FBG sensors, the wavelength data indicate that the settlement strain of the subgrade remains unstable and unconverted after the completion of subgrade filling. In contrast, after temperature compensation, the subgrade settlement deformation presents a stable and convergent state. This accurately reflects the actual filling process of the subgrade.

FBG settlement sensors were deployed synchronously throughout the project. One FBG settlement sensor on the same cross-section as the VI-FBG sensors was selected for comparative analysis, with a spacing of 2000 mm from the VI-FBG sensor. Figure 20 shows the sensor layout and the monitoring data of the FBG settlement sensor. The FBG settlement sensor directly measures the absolute subgrade displacement, whereas the VI-FBG sensor captures local subgrade compressive strain. Nevertheless, the two measurement curves exhibit broadly consistent trends, and both started to stabilize on 26 October 2025.

## 5. Conclusions

To meet the demand for high-precision vertical deformation monitoring of transportation infrastructure subgrades in complex geological areas such as mountainous regions, a miniaturized VI-FBG strain sensor encapsulated with GFRP and with a total height of 53 mm was developed in this study. A complete set of analyses, including structural optimization, performance testing, laboratory verification, and field engineering application, was completed, and the key conclusions are drawn as follows:

(1) Based on the Saint-Venant effect, the structure of the VI-FBG sensor was optimized. The encapsulated GFRP column of the sensor has a length of 38 mm and a diameter of 5 mm. The sensor exhibits good hysteresis performance, with a strain sensitivity of 1.2 pm/με and a temperature sensitivity of 16.84 pm/°C.

(2) Uniaxial compression test on a concrete specimen shows that the sensor can effectively measure the vertical strain, and the relative error between the measured strain and theoretical strain is controlled within 8% except for the first load level.

(3) The field monitoring experiment shows that with temperature compensation, the VI-FBG sensor is capable of effectively capturing subgrade local vertical compressive strain. Its time-history curve agrees well with the readings from the FBG settlement sensor, which can accurately reflect the deformation characteristics of the subgrade during the filling process.

In summary, the developed VI-FBG sensor is applicable to the measurement of local vertical compressive strain in subgrade. With GFRP encapsulation, it exhibits good durability and stable measurement performance. Future work will explore the vertical multi-point array application of VI-FBG sensors, investigate the compressive deformation of each layer in the process of layered subgrade filling, and conduct refined monitoring and evaluation of subgrade settlement by combining traditional settlement displacement monitoring data.

## Figures and Tables

**Figure 1:**
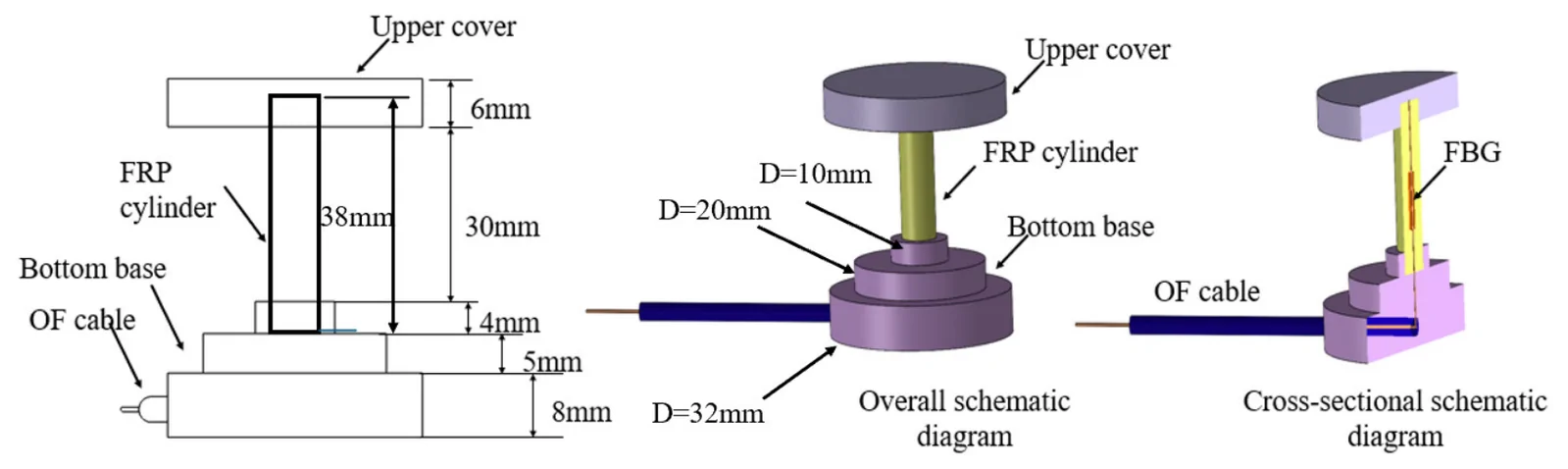
Structural schematic diagram of the VI-FBG sensor.

**Figure 2:**
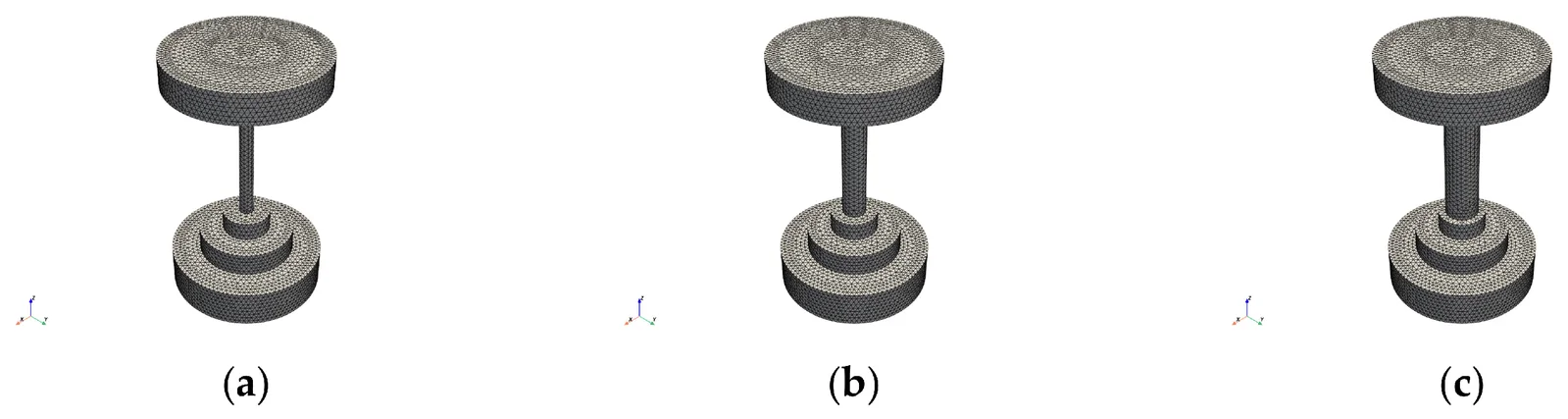
Finite element meshing: (**a**) 3 mm GFRP diameter, (**b**) 5 mm GFRP diameter, (**c**) 7 mm GFRP diameter.

**Figure 3:**
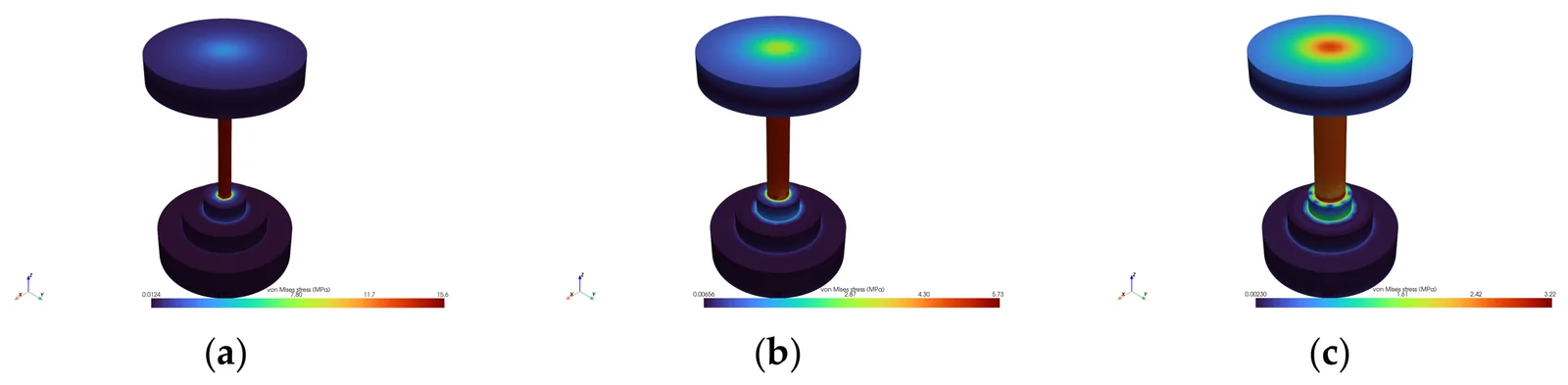
Overall von Mises stress contour: (**a**) 3 mm GFRP diameter, (**b**) 5 mm GFRP diameter, (**c**) 7 mm GFRP diameter.

**Figure 4:**
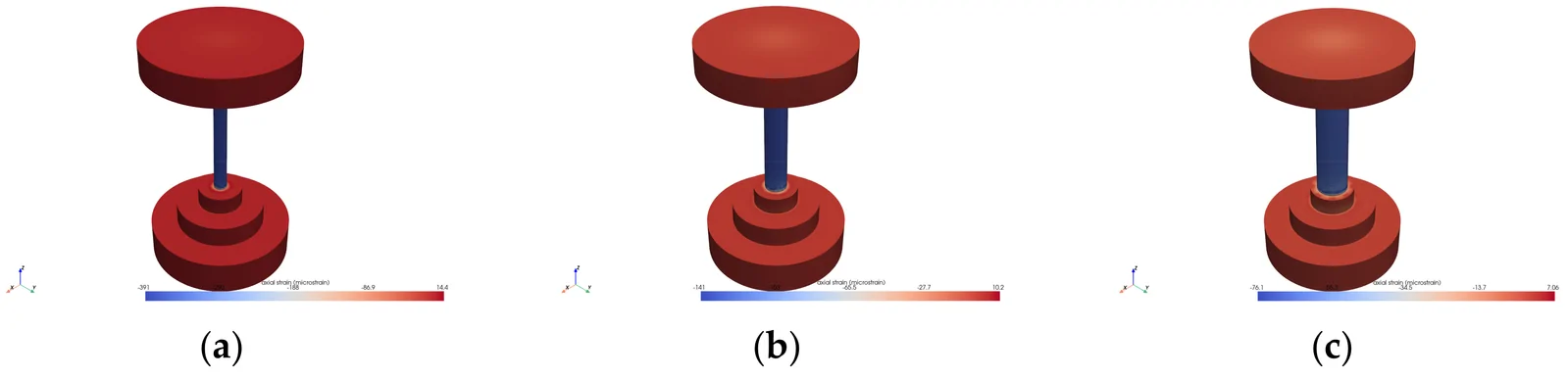
Maximum axial strain: (**a**) 3 mm GFRP diameter, (**b**) 5 mm GFRP diameter, (**c**) 7 mm GFRP diameter.

**Figure 5:**
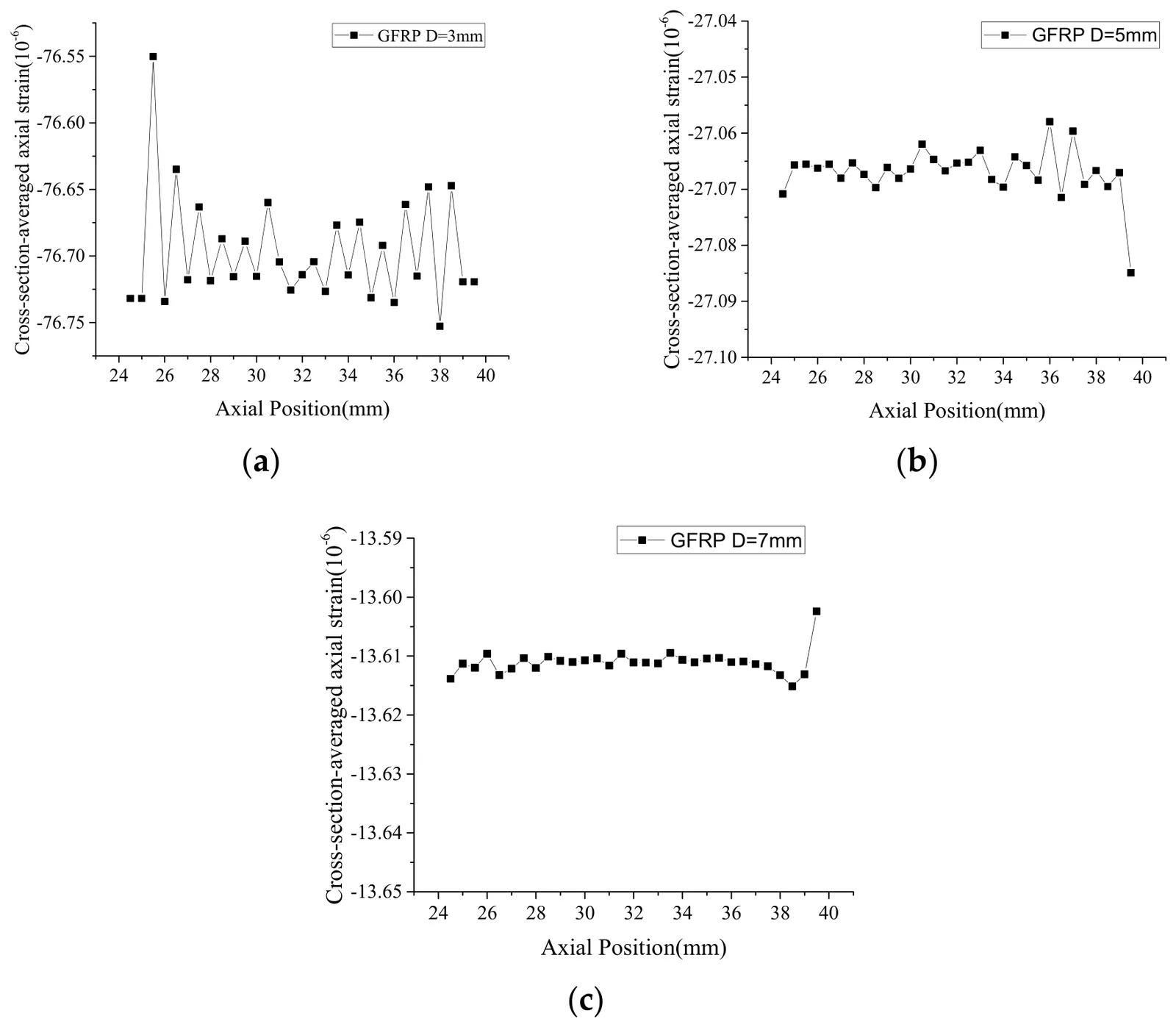
Axial strain distributed in the 15 mm FBG grating: (**a**) 3 mm GFRP diameter, (**b**) 5 mm GFRP diameter, (**c**) 7 mm GFRP diameter.

**Figure 6:**
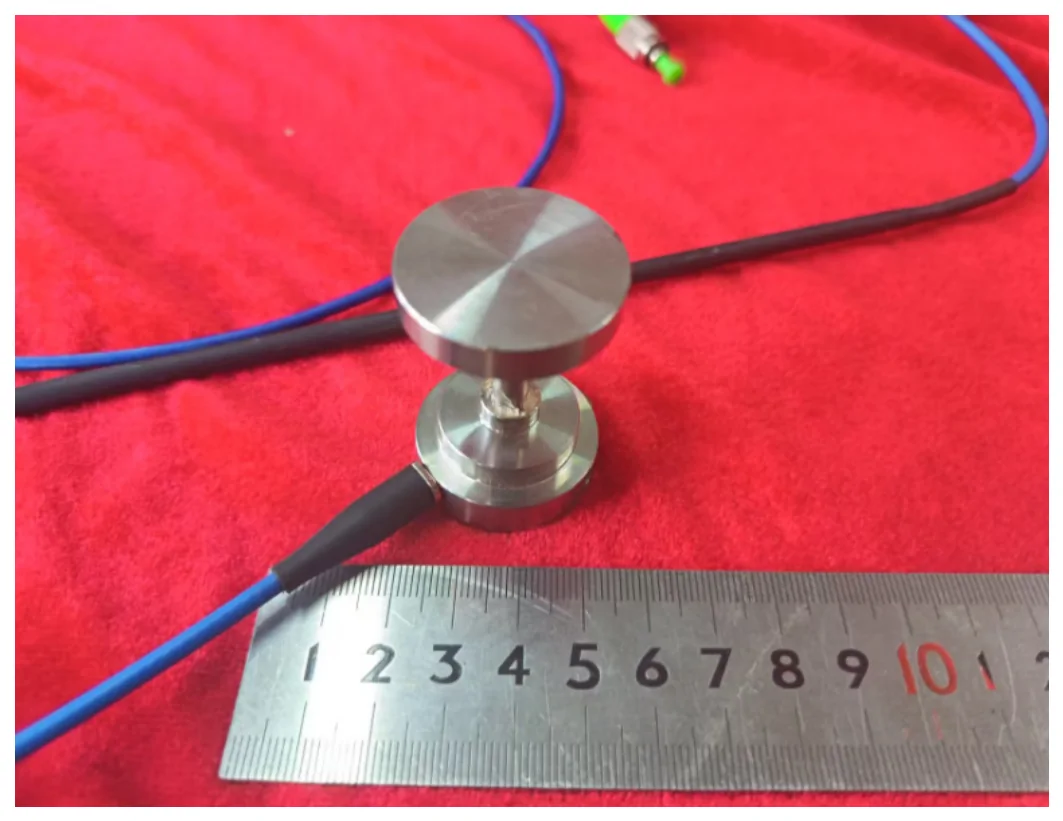
Physical drawing of the VI-FBG sensor.

**Figure 7:**
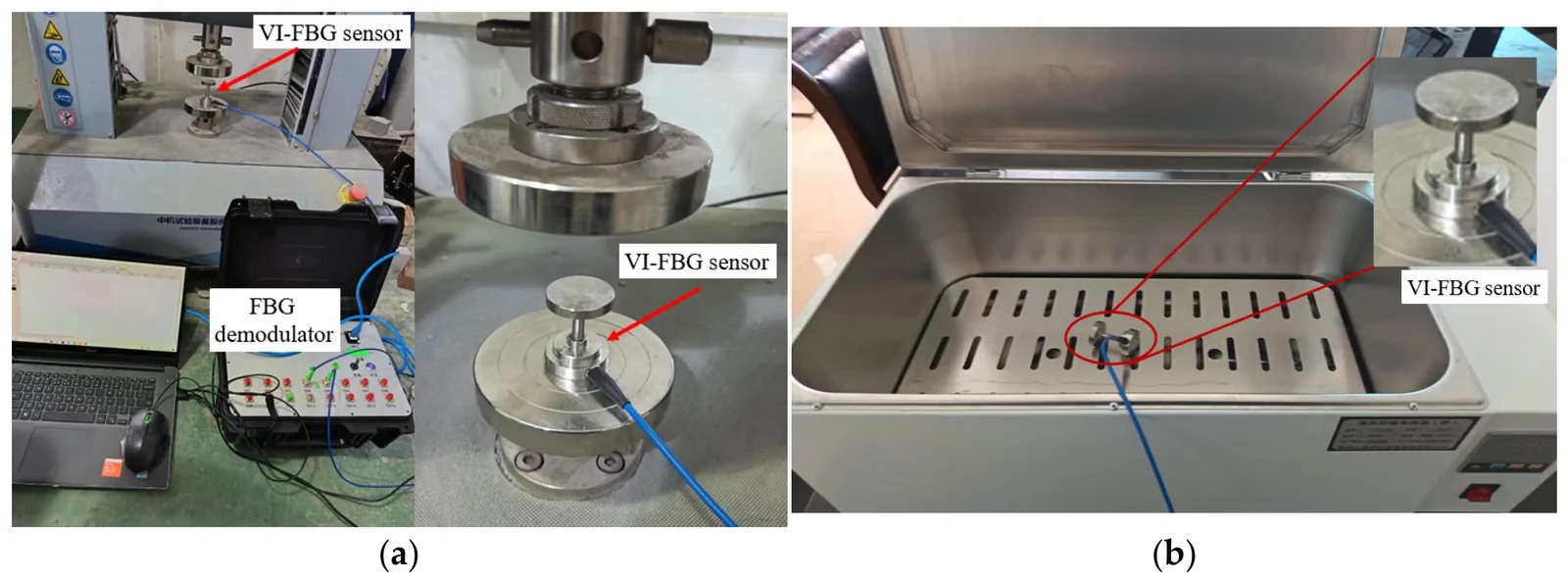
Test setup for strain and temperature sensing performance: (**a**) Strain test setup, (**b**) Temperature test setup.

**Figure 8:**
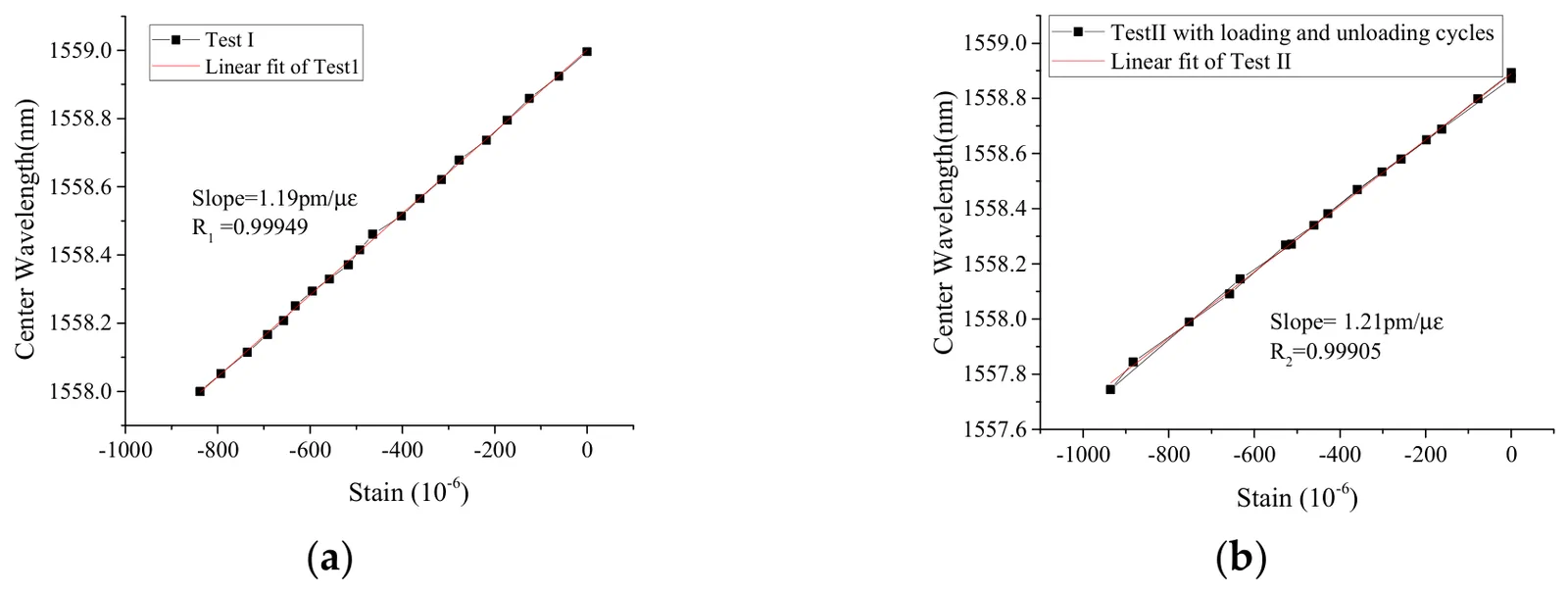
Test results of strain sensing performance: (**a**) Test I, (**b**) Test II with loading and unloading cycles.

**Figure 9:**
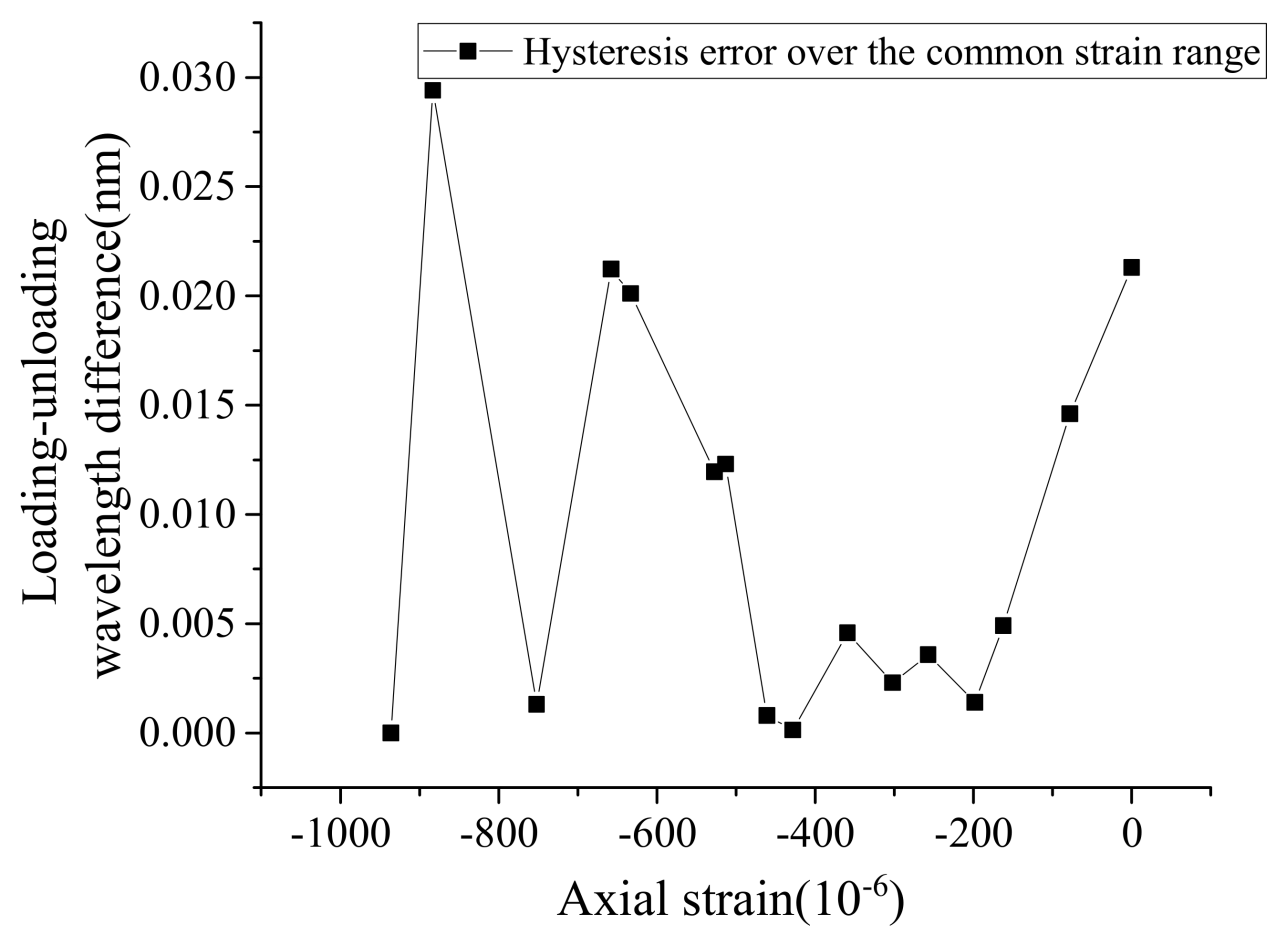
Hysteresis error curve of Test II.

**Figure 10:**
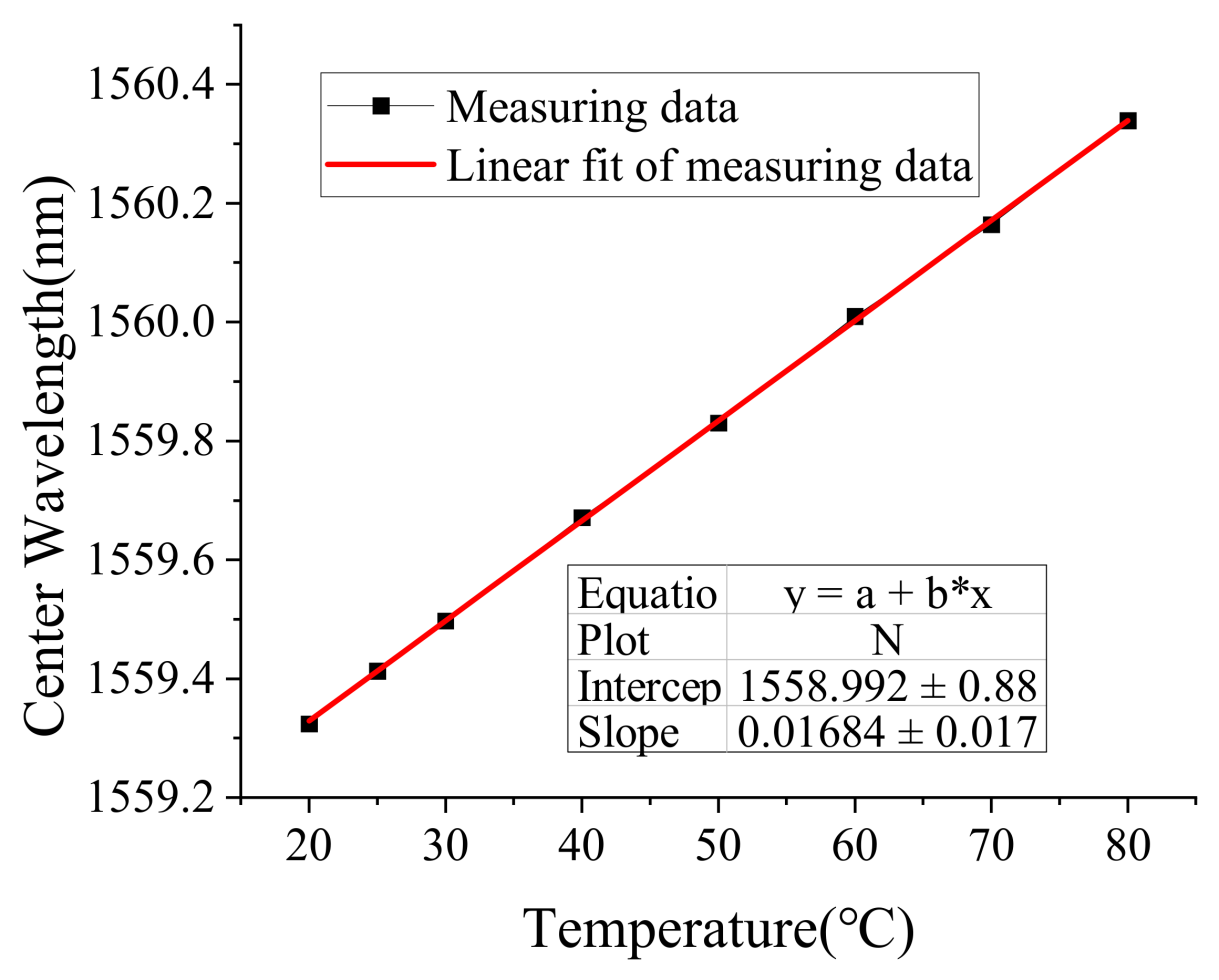
Test result of temperature sensing performance.

**Figure 11:**
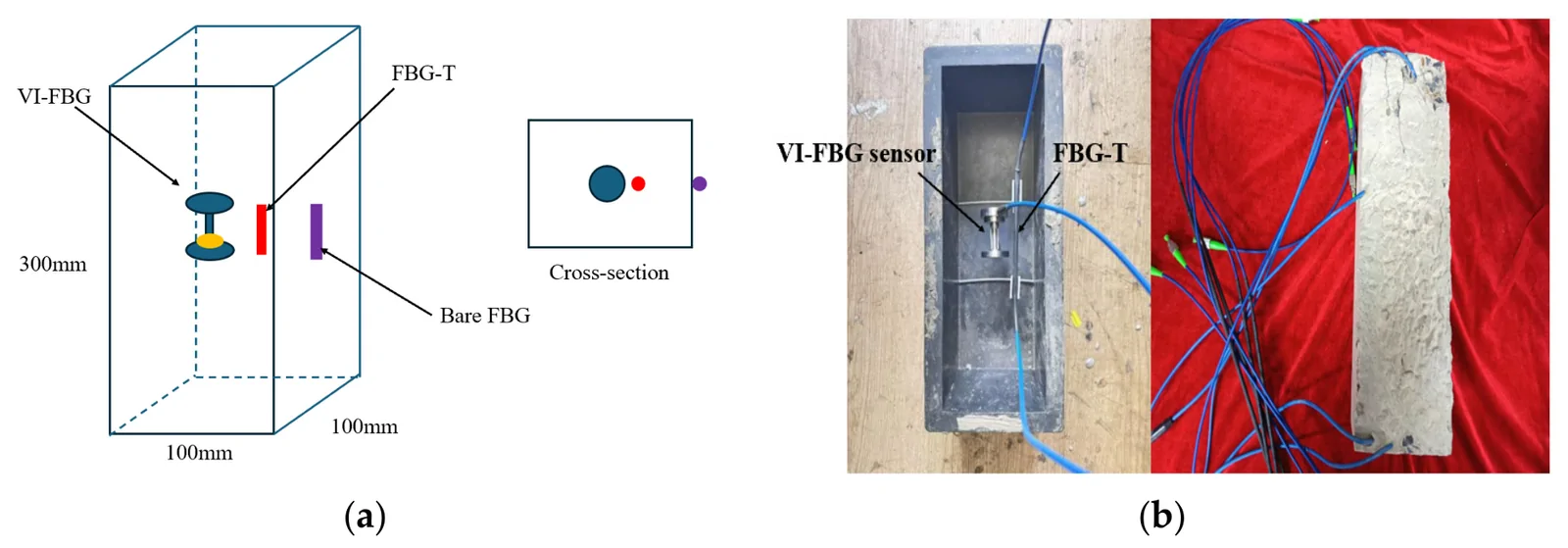
Sensor layout: (**a**) Schematic diagram of sensor layout, (**b**) photo of sensor arrangement.

**Figure 12:**
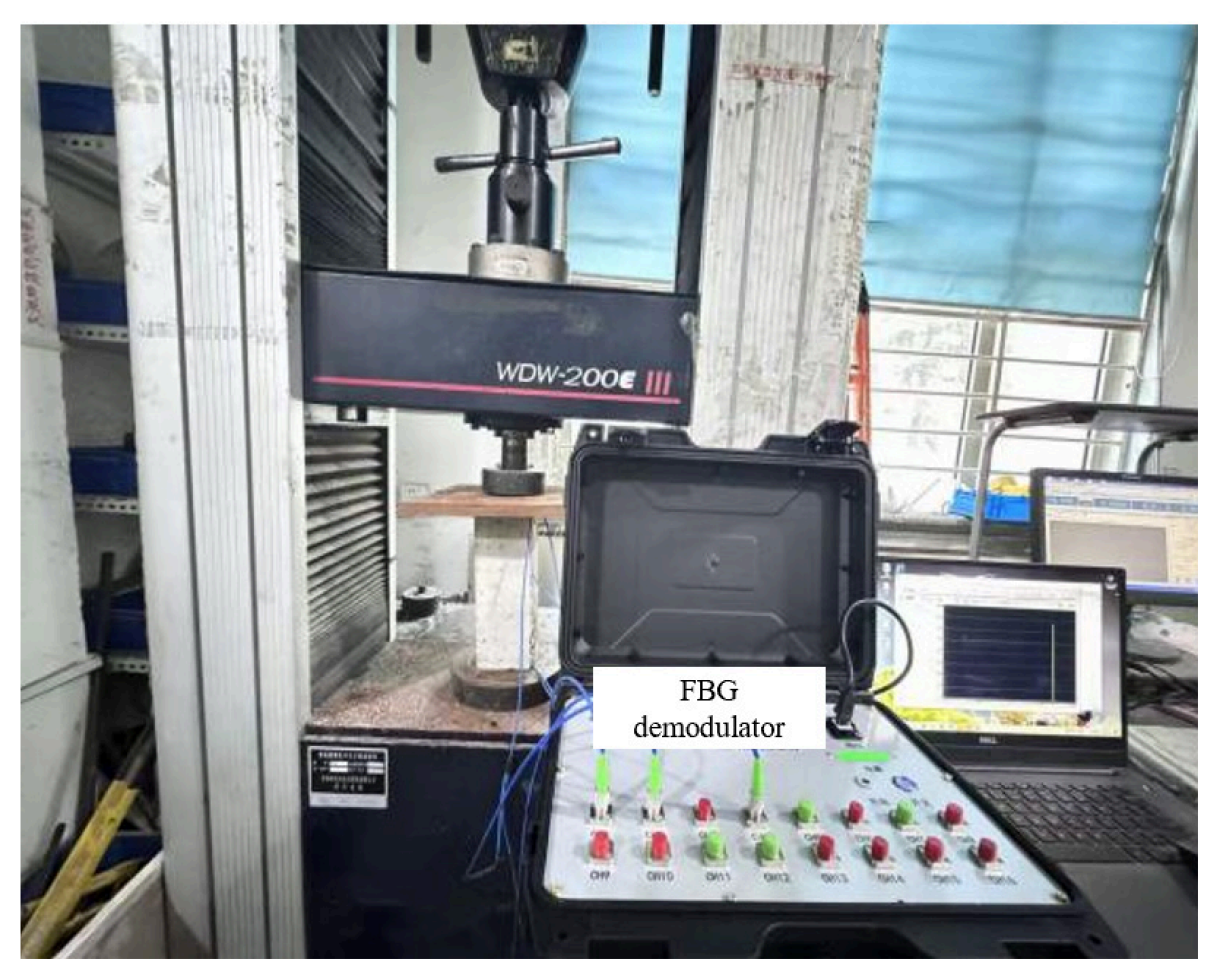
Concrete loading test system.

**Figure 13:**
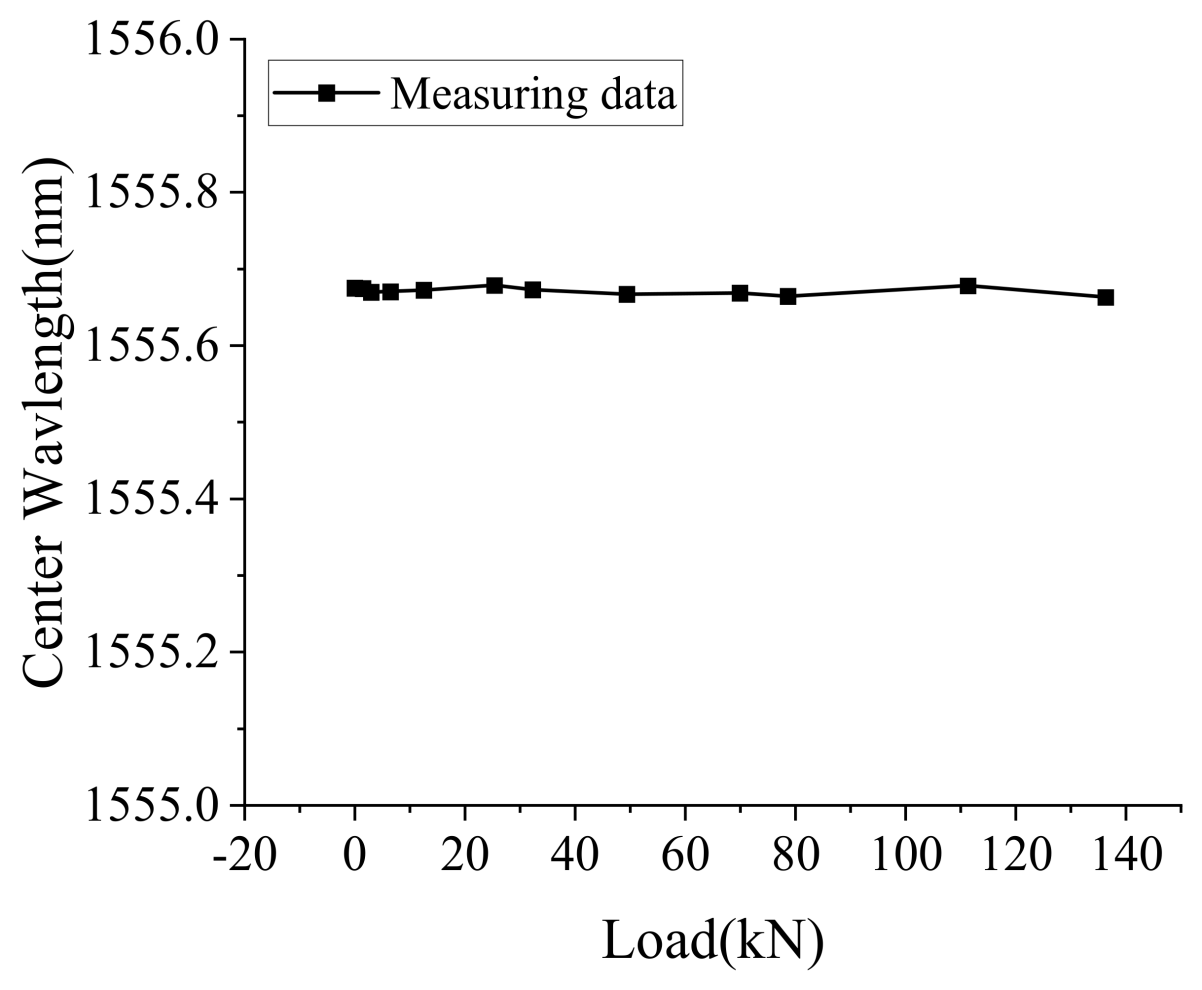
Wavelength change of FBG-T under loading.

**Figure 14:**
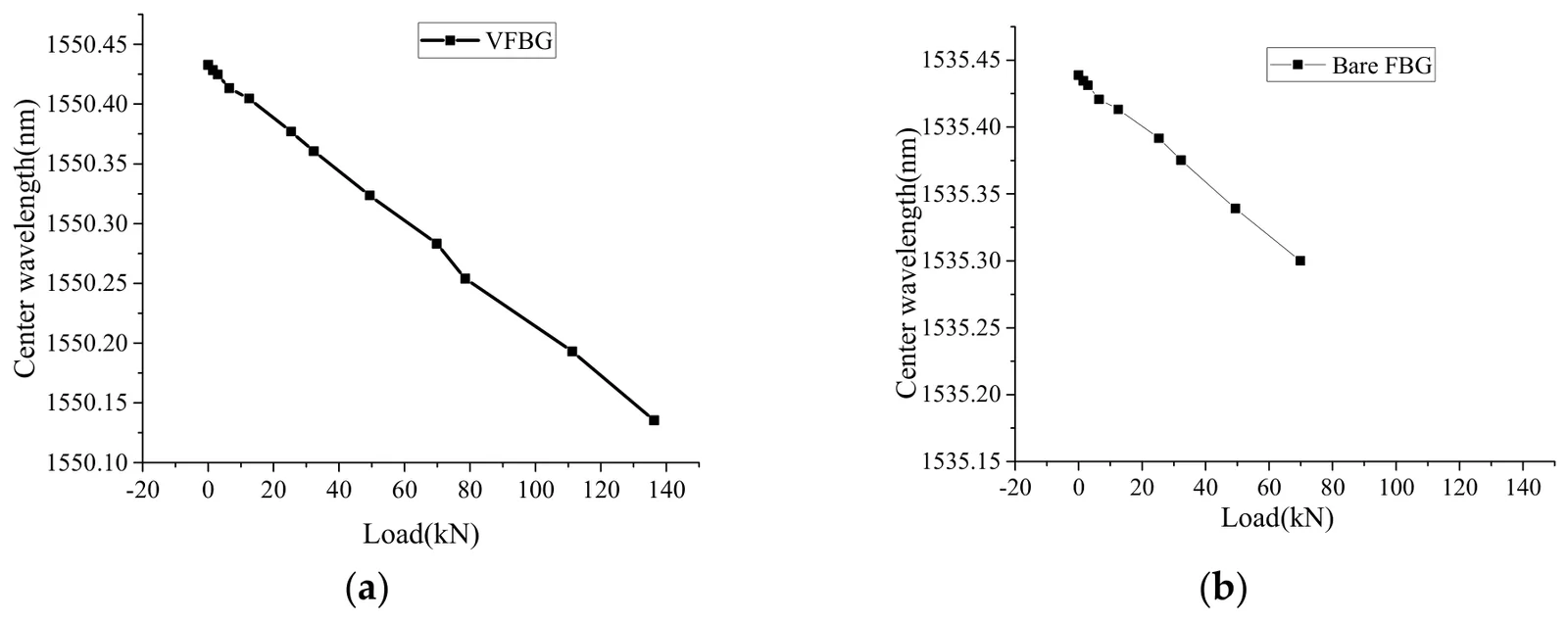
Wavelength change under loading: (**a**) VI-FBG, (**b**) Bare FBG.

**Figure 15:**
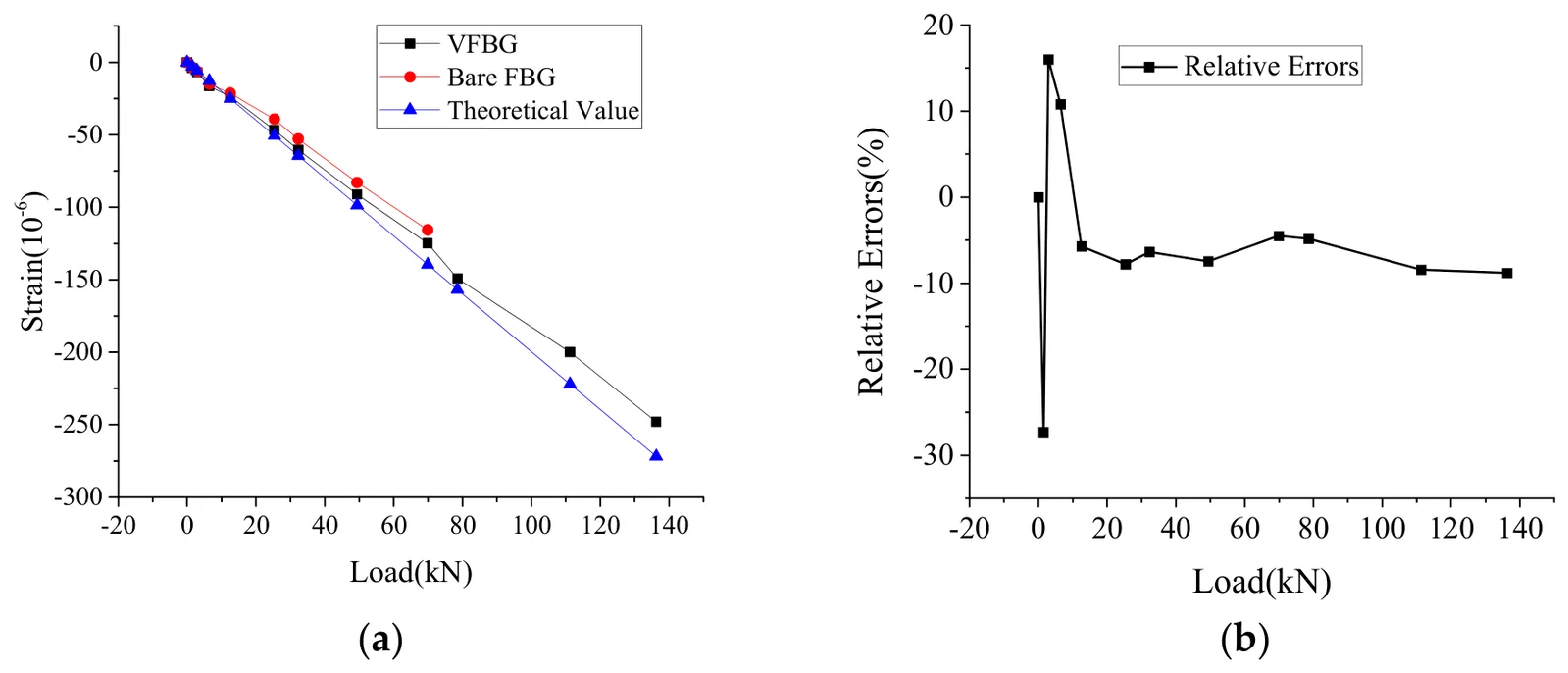
Comparison of measured and theoretical strain of C50 concrete specimen: (**a**) Variation curve of measured vertical strain, (**b**) Relative errors between the measured strain and theoretical strain.

**Figure 16:**
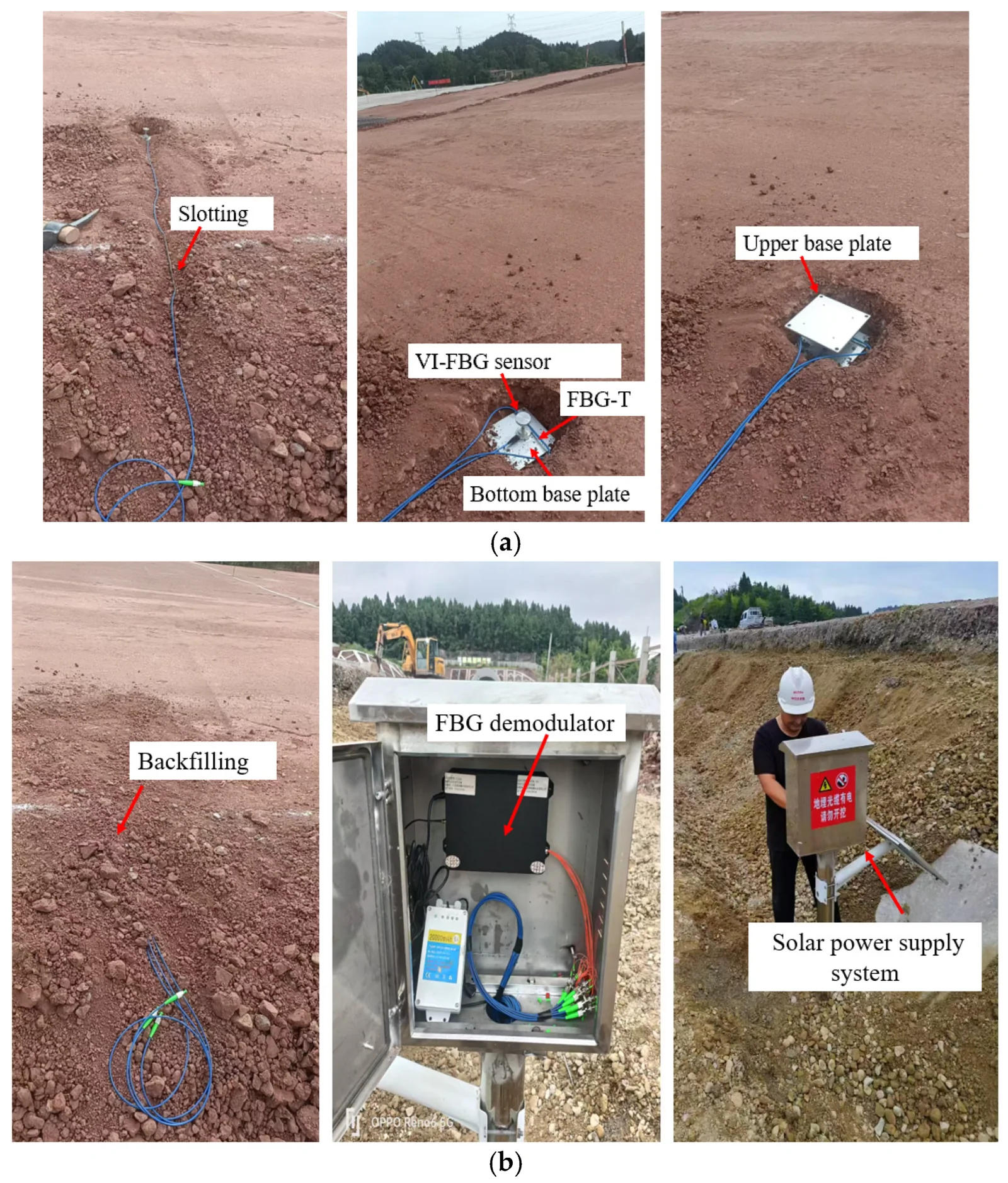
Sensor layout and test device for field monitoring test for high-speed railway subgrade: (**a**) Schematic diagram of on-site sensor layout at the bottom of the subgrade bed, (**b**) On-site layout of the solar power supply and data acquisition system.

**Figure 17:**
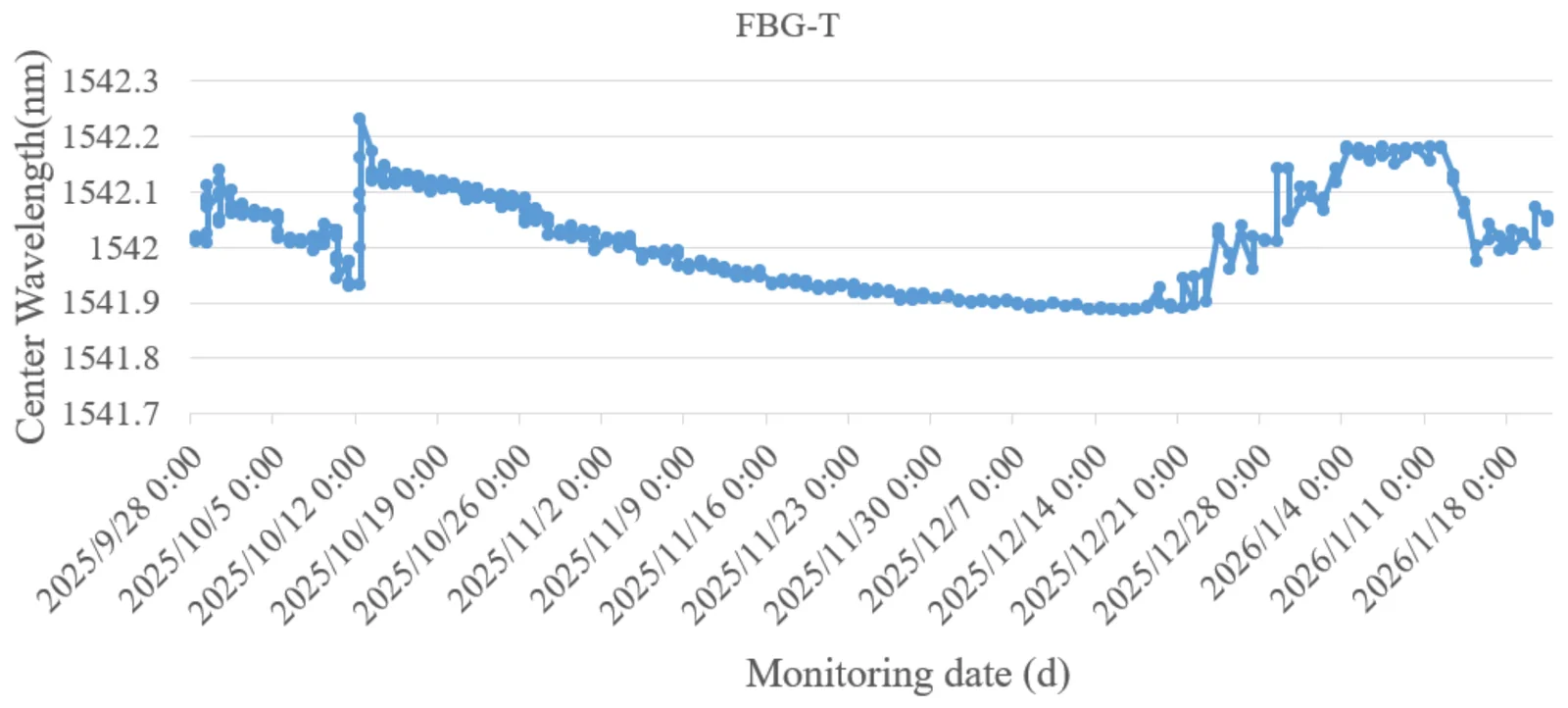
Wavelength variation curve induced by internal temperature changes in the subgrade.

**Figure 18:**
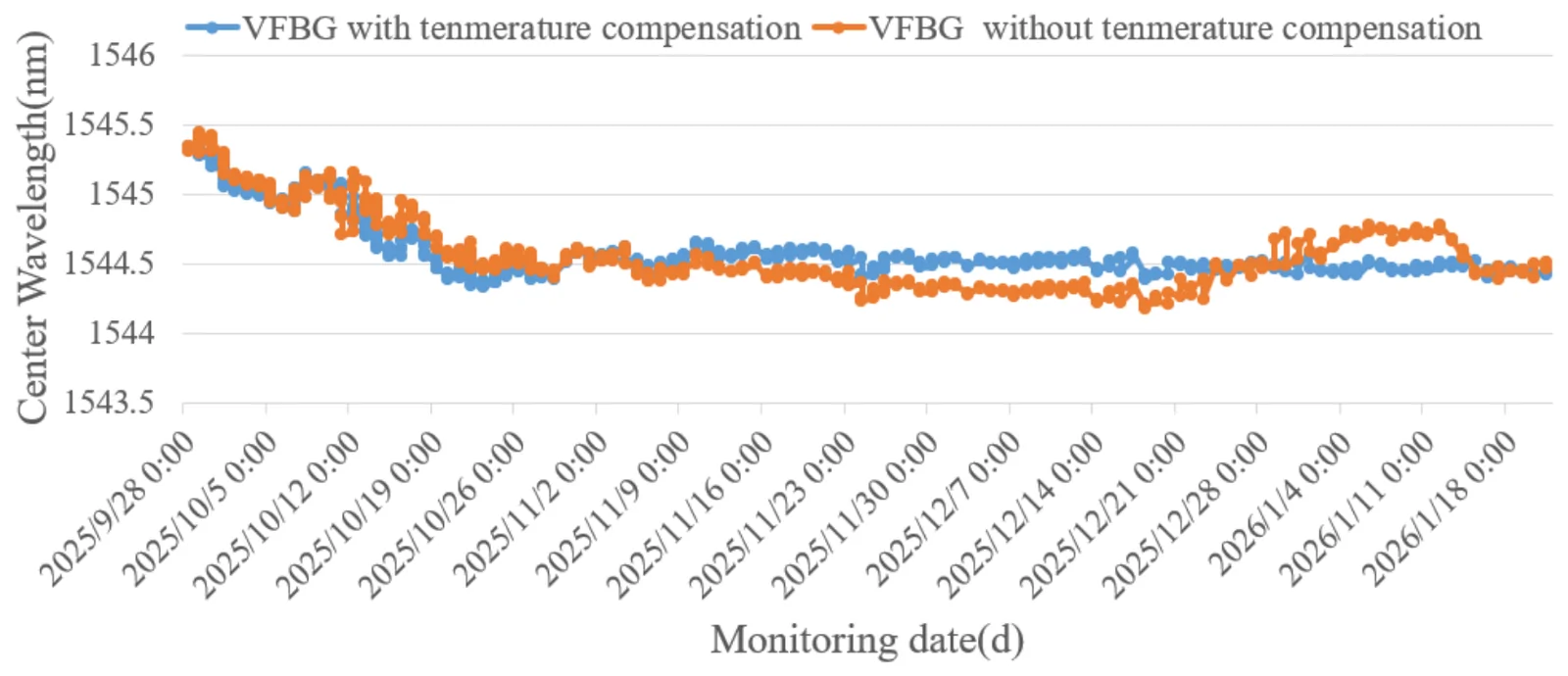
Wavelength variation curves of VI-FBG sensors before and after temperature compensation.

**Figure 19:**
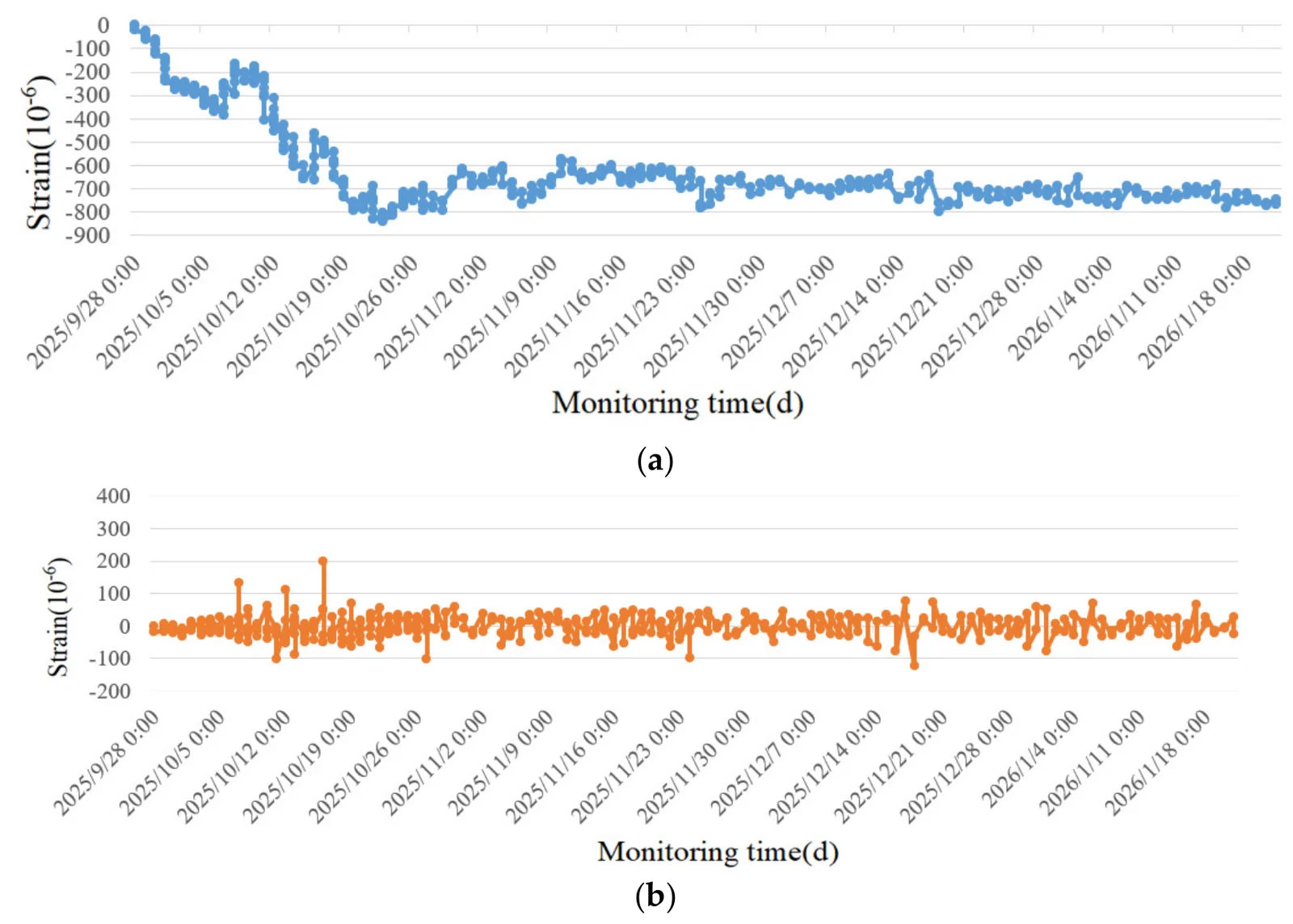
Vertical strain monitoring curves during the high-speed railway subgrade filling period: (**a**) Variation curve of cumulative vertical strain during the subgrade filling period, (**b**) Variation curve of single vertical strain during the subgrade filling period.

**Figure 20:**
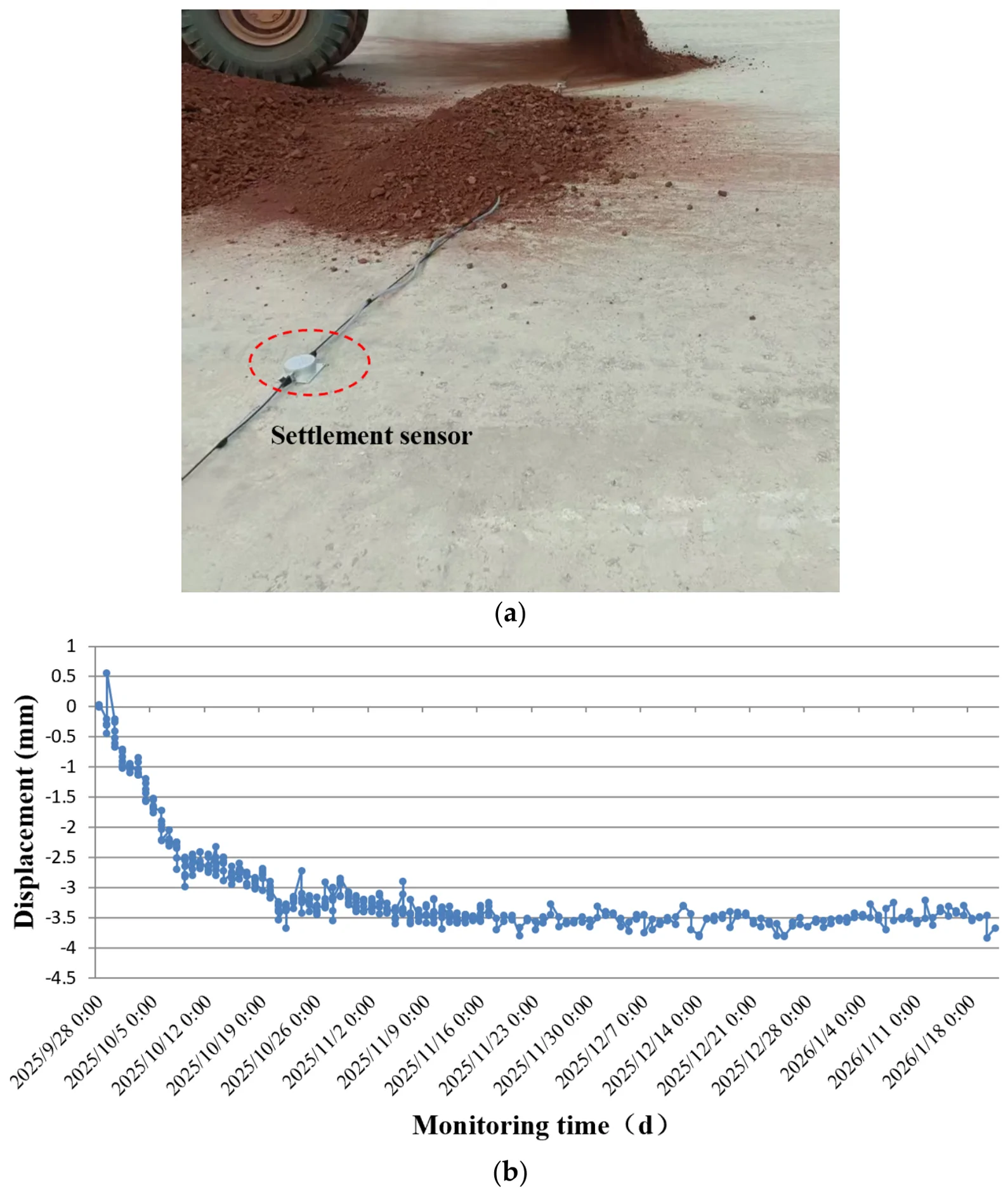
Subgrade settlement monitoring based on settlement sensor: (**a**) sensor layout, (**b**) displacement data.

## Data Availability

Data will be made available on request.

## References

[1] Tanikawa T., Hamada J., Yamashita K. (2025). Long-term settlement monitoring of a large building on soft ground using SAR satellite remote sensing. AIJ J. Technol. Des..

[2] Zhang X., Wang R., Liu X., Huang M., Huo L., Liu Y., Liu L., Liu H. (2025). Subway line settlement monitoring and analysis based on PS-InSAR technology and wavelet packet decomposition. Int. Arch. Photogramm. Remote Sens. Spat. Inf. Sci..

[3] Gutierrez F., Galve J.P., Lucha P. (2011). Integrating geomorphological mapping, trenching, InSAR and GPR for the identification and characterization of sinkholes: A review and application in the mantled evaporite karst of the Ebro Valley. Geomorphology.

[4] Lee J.S., Jeong S.H., Park G., Kim Y.S., Tubulure E., Kim S.Y. (2025). Geotechnical application of unmanned aerial vehicle (UAV) for estimation of ground settlement after filling and compaction. Transp. Geotech..

[5] Lu D., Liu J., Wang M., Gu S. (2025). State-of-the-art design and optimization of strain gauge-type load–displacement transducer for in situ nanoindentation systems. Sensors.

[6] Wu Z.H. (2025). Design of Multi-Point Displacement Strain Gauge and Its Monitoring System Based on Fiber Bragg Grating. Master’s thesis.

[7] Liu J.Q., Wan L., Xiao X.L., Bao T.F., Deng Y.Q. (2017). Analysis of monitoring data of multi-point extensometer for intake slope in Yantan Expansion Project. J. China Three Gorges Univ. (Nat. Sci.).

[8] Li W., Zhang F. (2016). Application of multi-point extensometer in foundation settlement monitoring of Bagatelle Dam in Mauritius. Sichuan Water Power.

[9] Markom A.M., Saharudin S., Hisham M.H. (2025). Systematic review of fiber-optic distributed acoustic sensing: Advancements, applications, and challenges. Opt. Fiber Technol..

[10] Moffat R.A., Beltran J.F., Herrera R. (2015). Applications of BOTDR fiber optics to the monitoring of underground structures. Geomech. Eng..

[11] Biondi A.M., Zhou J., Guo X., Wu R., Tang Q.X., Gandhi H., Yu T.Z., Gopalan B., Hanna T., Ivey J. (2022). Pipeline structural health monitoring using distributed fiber optic sensing textile. Opt. Fiber Technol..

[12] Wu R., Biondi A., Cao L., Gandhi H., Abedin S., Cui G., Yu T., Wang X. (2023). Composite bridge girders structure health monitoring based on the distributed fiber sensing textile. Sensors.

[13] Zheng Y., Li Z., Yu J., Xiao J. (2022). Experimental research on a novel spring-shaped fiber-optic displacement sensor for settlement monitoring. Measurement.

[14] Zhu H.-H., Gao Y.-X., Chen D.-D., Cheng G. (2024). Interfacial behavior of soil-embedded fiber optic cables with micro-anchors for distributed strain sensing. Acta Geotech..

[15] Zhang D., He J., Xue Y. (2019). Investigation of settlement monitoring method based on distributed Brillouin fiber optical sensor. Measurement.

[16] Shi B., Zhang D., Zhu H.H., Zhang C.C., Gu K., Sang H.W., Han H.M., Sun M.Y., Liu J. (2021). DFOS applications to geo-engineering monitoring. Photonic Sens..

[17] Wang H.G., Gong H.L., Zhang J.C., Zhu L., Ning D., Zhou C.F., Yan X.G. (2025). Layered monitoring of ground subsidence based on ultra-weak FBG sensing technology: A case study in Gaoyang County, China. Micromachines.

[18] Fang K., Tan D.-Y., Zhang C.-C., Shi S.-G., Sang H.-W., Shi B. (2025). Nature-based profiling of subsurface soil stiffness driven by tidal forces. Geophys. Res. Lett..

[19] He J., Xue Y., Xu J., Zhang D., Zhang S. (2020). Whole-process monitoring of sinkhole collapse based on distributed optical fiber strain-vibration joint system and its case study in railway subgrade. Opt. Fiber Technol..

[20] Zhou Z., Liu W., Huang Y., Wang H., He J., Huang M., Ou J. (2012). Optical fiber Bragg grating sensor assembly for 3D strain monitoring and its case study in highway pavement. Mech. Syst. Signal Process..

[21] Liu B., Zhang S.H. (2019). Deformation measurement of glass structure using FBG sensor. Photonic Sens..

[22] Hill K.O., Malo B., Bilodeau F. (1993). Bragg grating fabricated in monomode photosensitive optical fiber by UV exposure through a phase mask. Appl. Phys. Lett..

